# Strategies and goals in Emotion Regulation models: a systematic review

**DOI:** 10.3389/fpsyg.2024.1425465

**Published:** 2024-10-02

**Authors:** Consuelo Martínez-Priego, Belén Poveda García-Noblejas, Pablo Roca

**Affiliations:** Department of Psychology, Faculty of Health Sciences, Universidad Villanueva, Madrid, Spain

**Keywords:** emotion regulation, strategies, goals, models, theories, frameworks, wellbeing

## Abstract

**Introduction:**

Studies examining the role of Emotion Regulation (ER) do not consistently explain the underlying model or theory they are employing, resulting in a conflation of different strategies and goals within the ER scientific literature. This study aims to conduct a systematic review and conceptual analysis of the primary strategies and goals advocated in the ER models, theories, and frameworks. Furthermore, we explored the distinctions between the prevailing contemporary ER models and classical conceptions of emotional dynamics, such as those proposed by Aristotle, Descartes, and Darwin.

**Methods:**

An electronic search was conducted in the Web of Science, Medline, and Scopus databases in November 2023. The key search terms used were grouped into two different topics: Emotion Regulation and Models/Theories/Frameworks. Articles were included if they reported one or more ER model in healthy individuals or emotionally disordered individuals and if they were published in a peer-reviewed journal in English in the last 5 years (from 2019 to 2023). A total of two reviewers independently assessed the titles, abstracts, and full texts. Models identified were summarized and classified based on the different ER strategies and goals.

**Results:**

Of the 1,012 titles for initial consideration, 139 articles met the full eligibility criteria and were included for data extraction and synthesis. The review identified 10 ER models, and the most commonly used were the Process Model of Emotion Regulation and the Difficulties in Emotion Regulation. There was a great deal of homogeneity among the proposed ER strategies and goals: the cognitive dimension is the core of ER strategy, and the ER goals are primarily hedonic or instrumental in nature.

**Discussion:**

Both Descartes and Darwin views were present in the ER models; however, some of the most significant contributions in Aristotelian proposal seem to be forgotten, such as the integration of the physical, operational, and growth dimensions (eudaimonic goals).

**Systematic review registration:**

This systematic review was conducted in accordance with the PRISMA guidelines and was preregistered at Prospero platform (CRD42023491948).

## Introduction

In recent decades, Emotion Regulation (ER) has emerged as a pivotal concept within the field of psychology, garnering substantial attention from researchers across various subfields (clinical, educational, organizational, etc.). Moreover, the study of ER has expanded beyond traditional psychological domains, encompassing interdisciplinary perspectives from fields such as psychiatry, neuroscience, or social context (Grecucci et al., 2017; Rodriguez-Moreno et al., 2022). ER has become increasingly recognized as fundamental to adaptive functioning and psychological wellbeing (Sheppes et al., 2015; Kraiss et al., 2020), being a key active ingredient in contemporary psychological interventions (e.g., Rojas et al., 2023; Roca et al., 2021).

Usually defined as the ability to monitor, evaluate, and modify emotions to attain a goal (Thompson, 1994), ER encompasses a spectrum of strategies individuals use to modulate their emotional experiences (e.g., cognitive reappraisal, suppression, acceptance, etc.), as well as diverse array of goals individuals seek to accomplish through these regulatory efforts (e.g., enhancing wellbeing, maintaining social relationships, meet a challenge, etc.) (Kopp, 1989; Tamir et al., 2007). However, studies examining the role of ER do not consistently explain the underlying model or theory they are employing, resulting in a conflation of different strategies and goals within the ER scientific literature (Sutton, 2004; Moumne et al., 2021). Without a clear conceptual foundation of the different strategies and goals employed in the main ER models and theories, researchers may struggle to interpret findings, compare results across studies, and identify gaps in the literature (Tull and Aldao, 2015).

Over the years, different models of ER have emphasized various specific strategies as either adaptive or maladaptive, and as potential risk or protective factors against psychopathology (Aldao et al., 2010; Naragon-Gainey et al., 2017). Adaptive ER strategies, such as cognitive reappraisal, acceptance, or problem-solving, involve actively modifying one’s interpretation of emotional stimuli or addressing the underlying cause of distress, and are associated with better psychological wellbeing, reduced psychopathology, and positive affect. Maladaptive ER strategies, such as avoidance, suppression, or rumination, entail ineffective or counterproductive attempts to regulate emotions, often results in exacerbation of psychopathological symptoms and negative affect (Gross et al., 2019). However, in line with contemporary ER models (e.g., ER flexibility), there is no clear correspondence between the use of specific strategies and their adaptive value, and it depends on the specific regulatory goals prompting the use of ER in each given situation (Boemo et al., 2022).

Emotion regulation is a motivated process, so the strategies and outcomes of ER are contingent upon the goals it seeks to fulfill, and these goals/motives are crucial to understanding the multifaceted nature of ER processes (Tamir et al., 2020). Attempts to regulate emotions, whether by upregulating or downregulating specific affective states, can be driven by different goals and may lead to varied consequences depending on the context (Aldao et al., 2015; Ford et al., 2019). The ability to regulate emotions effectively according to ongoing goals and contextual demands is central to various aspects of psychosocial functioning, including achieving specific outcomes, maintaining social relationships, and enhancing wellbeing. For instance, Eisenberg et al. (2004) underscored the importance of emotion regulation in social functioning, highlighting its role in navigating social interactions and relationships. Brackett et al. (2010) emphasized its significance in academic and work performance, suggesting that effective ER can positively impact productivity and achievement. In fact, there is an association between ER strategies and goals: for instance, reappraisal of emotional experience is crucial for hedonic goals, whereas expressive suppression is important for social goals (Wilms et al., 2020). Importantly, ER goals are not static but can vary based on contextual demands and individual differences (Koval et al., 2023).

Despite the importance of these two concepts, most studies lack a critical categorization of how (i.e., strategies) and why (i.e., goals) people regulate their emotions, and as far as we know, no studies to date have reviewed the main ER strategies and goals across the different models and theories used in scientific literature. Therefore, this study aims to conduct a systematic review and conceptual analysis of the primary strategies and goals advocated in current scientific literature on Emotion Regulation Models, theories, and frameworks. The categorization of strategies and goals will be carried out based on the understanding of emotion as a process, as a multidimensional response, and as a state. Additionally, we conduct a critical analysis of the emotional regulation models identified in the scientific literature, comparing them with the classical theories of Aristotle, Descartes, and Darwin, which have had a significant influence on research on emotion regulation. We examine the new elements that have been incorporated and whether these emotion regulation models have a solid theoretical and conceptual foundation that guides the interpretation of results. The review aims to address the following questions: (1) what models, theories, and frameworks have been employed to elucidate Emotion Regulation in the current scientific literature? (2) What strategies and goals do these models of emotional regulation promote? (3) What differences exist concerning the modern emotional regulation models and the classical conceptions of emotional dynamics (i.e., Aristotle, Descartes, and Darwin)?

## Theoretical framework

We compared contemporary ER models identified in the review with the three classic proposals that have inspired emotion theories throughout the history of psychology (Carpintero, 2003): the views of Aristotle, Descartes, and Darwin. Each of them contributes a concept of emotion, of psychological dynamics (thus, of the places where ER is possible), and of the goals of this dynamics. Aristotle was the first author to analyze emotion from the perspective of the three-response system prevalent in contemporary models (i.e., cognitive, biological, and behavioral) (Lyons, 1985). Descartes, on the other hand, introduced the dual pathway (behavioral and cognitive) that gave rise to the two classical psychological schools of thought: cognitivism and behaviorism (Martínez-Priego, 2012; Malo Pe, 2004). Finally, Darwin contributed to the study of emotions by emphasizing the importance of phylogenesis, leading to a paradigm shift in the understanding of the concept of emotion (Fernández-Berrocal, 2009).

The concept (not the term) of ER has a long history dating back to Aristotle. This author (biologist and philosopher) proposes a multidimensional explanation of emotions, encompassing cognitive, physiological, and behavioral dimensions. Specifically, in the Rhetoric (Aristotle, 1985 [1378a]), the significance of evaluative words (cognitive dimension of emotion) is the trigger for emotional response, as in the case of an insult that triggers the emotion of anger. In the De Anima (Aristotle, 1984 [403a]), the explanation of feelings and passions occurs within the context of the operations of the living being. The human being also performs operations such as remembering, valuing, coping, etc., all thanks to their organic faculties, so Aristotle understands that emotions have a physiological basis (Davis and Panksepp, 2018; Martínez-Priego, 2012). This explanation is further elaborated in the Nicomachean Ethics (Aristotle, 2018 [1110a, 1111b]), where Aristotle distinguishes between voluntary and involuntary actions. Passions (fear, pain, regret, anger, courage, etc.) are factors that influence the distinction between these types of actions. These feelings can affect the involuntary nature of acts, but they can also be integrated into voluntary acts, facilitating the action itself (Aristotle, 2018 [1104b]). This is a model of the role of emotion in decision-making (Solomon, 1993; Phelps et al., 2014). Aristotle also presents an organizing element of affective dynamics: human growth. Indeed, all human activity is oriented toward eudaimonia, that is, happiness involving harmony of the psyche and virtuous life (not hedonic wellbeing). As is well known, virtue is not a terminal point in the biographical process but a middle ground between negative extremes for the individual (Aristotle, 2018 [1106b]). In summary, in Aristotle, cognition (intelligence), physiology (organic faculties), and action (will) (Martínez-Priego and Romero-Iribas, 2024) are the resources/strategies for ER; whereas the goal of ER is to achieve a life adjusted to reality, happy or virtuous; a life oriented toward human growth.

Descartes marked a turning point in the history of psychology by inaugurating, in a scientific manner, the distinction and separation between the soul (mind) and the body (Carpintero, 2003). This separation persists in proposals such as James (1884) or in psychological schools that omit one of the two elements, such as the mind in the case of behaviorism (Skinner, 1974). For Descartes, passions are obscure and confused ideas of the soul caused by the body, meaning they have some organic basis. Within the Cartesian model, these passions can also be termed feelings or emotions of the soul (Descartes, 1997, arts. 27–29). They are the ones that most deeply affect the soul, which is why passions are regarded with suspicion, though not inherently negatively. Within the causal relationship between body and soul (principle of emotions), we must highlight the causal relationship (control) of the will over the soul and the body, and therefore its control over the passions (Descartes, 1997, art. 18). Ultimately, Descartes establishes “control” (of knowledge or behavior) exercised by the will (mind) as a strategy of ER. The goal of this ER strategy is to make emotions useful to the body (survival and satisfaction) and the soul (so that it can reach the highest passion, which is love) (Descartes, 1997, arts. 137–139).

Later, Darwin introduced a new biological-anthropological framework for ER, marking a milestone in the history of modern science (Carpintero, 2003). Evolution signifies an increase in complexity among species, but not a qualitative difference between them. The struggle for survival and natural selection aims to adapt species to their environment (Darwin, 1854). Indeed, emotions have adaptive significance, representing the appropriate response to environmental stimuli which, as formulated years later, serve the triple adaptive, communicative, and motivational function (Frijda and Mesquita, 1994). Darwin asserts the qualitative identity between animal and human psychological dynamics (Darwin, 1872), meaning we share sensations, impressions, and emotions, although in the case of humans, they are more complex. Similarly, strategies of ER are analogous and determined by instincts. Lastly, the goal of ER is adaptation, in other words, internal and contextual homeostasis.

### Emotion: a multidimensional construct

Different models of ER rely on the construct of “emotion” (Scherer, 1982; Tull and Aldao, 2015). Although there is a wide variety of “Emotion Theories” (Plutchik, 1980; Kleinginna and Kleinginna, 1981), there is consensus that emotions can be understood as: (1) the study of the triple response system: cognitive, physiological, and behavioral (Lyons, 1985; King, 2020); (2) the study of three moments: the evaluation-valuation of the stimulus, the neuroendocrine activation in response to the stimulus, and the multidimensional manifestation of the emotion (Scherer, 1982; Fernández-Abascal et al., 2010); and (3) Additionally, considering the subject and not just the construct, emotions can be defined as “states of the subject” concomitant with cognitive-evaluative (appraisal) and tendential operations (Polo, 2015a; Martínez-Priego, 2010), that is, those that drive the subject to action (Aspinwall and Taylor, 1997). Emotions are not acts, but companions of acts. In this third sense, we can intervene on the acts and not on the emotions themselves that accompany them.

While emotion as a process offers opportunities to explain how cultural context and individual differences (personality and life experiences) affect emotional states (Arnold, 1960; Komulainen et al., 2014), it also allows for viewing emotion as a psychosomatic reality by integrating the three moments of the emotional process (i.e., evaluation-valuation of the stimulus, the neuroendocrine activation in response to the stimulus, and the multidimensional manifestation of the emotion) (Rof Carballo, 1952, 1961); that is, it does not necessarily imply a view in which mind–body are juxtaposed. In such a case, it can be argued that the Emotional Brain (Rof Carballo, 1952; LeDoux, 1999) is the organ of emotion and integrates cognitive and coping-tendential aspects. Thus, the emotion construct delimits the areas where modification of the emotional process and paths for achieving better ER can be opened. Thus, emotion, whether understood as a response, process, or state, allows for identifying the different ways in which the emotional process can be modified and promote ER: changes in the triple response system, changes in the three temporal moments of the emotional process, or changes in the acts that accompany emotional states. The question of strategies and goals of ER remains to be clarified.

### Strategies and goals in ER

Empirical evidence suggests that ER occurs through: (1) cognitive processes (Naragon-Gainey et al., 2017; Sutton, 2004; Gross, 2015): such as reappraisal, rumination, or distraction; (2) motivational-coping processes (Antaki and Brewin, 1982; Crespo Suárez, 1982): such as setting meaningful goals or impulse control; and (3) behavioral processes (Scherer, 1982): such as seeking social support or problem-solving. This triple pathway (cognitive, motivational-coping and behavioral) can serve as a criterion for the classification of ER strategies. Furthermore, the neuroendocrine activation processes (Etkin et al., 2015; Messina et al., 2021) would be transversal to the three previous dimensions (e.g., there is brain activation during cognitive, motivational, and behavioral processes), and therefore, is less useful for discriminating and categorizing the different ER strategies. However, it is crucial to ask whether the ER strategy precedes the goal or vice versa. According to Aldao and Tull (2015), ER outcomes focus on changes in subjective experience (feeling), neuroendocrine activation, and expressive behavior. All these changes are, for the subject, subsequent in time to the ER strategy. However, if beliefs or implicit theories of various kinds are included in equation (Tamir et al., 2007; De Castella et al., 2013; Moumne et al., 2021), it turns out that these beliefs come first in time, and they are the goals of ER that conditions the strategies used, whether consciously or unconsciously.

Some studies suggest that hedonic and contra-hedonic goals can be distinguished in ER (Webb et al., 2012), considering that contra-hedonic ones are “suffered” to achieve other goals of greater interest to the subject. Something similar is concluded from Tamir (2009) study when seeking the reasons why people want to feel a certain type of emotion (positive or negative) and why. Ultimately, a contemporary taxonomy distinguishes between two different ER motives (Tamir, 2016): (1) hedonic goals, aim at changing the current pleasure-to-pain ratio by approaching pleasure and avoiding pain; and (2) instrumental goals (i.e., contra-hedonic nature), that target other potential benefits of emotions, such as creating and maintaining positive social relationships.

It is surprising that these two types of ER goals are established in the scientific literature (i.e., hedonic, and instrumental). It is known that the hedonic goal has an opposite: the eudaimonic goals (happiness as virtue, good life, or flourishing), while de contrary is the contra-hedonic (instrumental). However, if we adhere to a classification of emotions that considers the trigger and the target of the emotion, these can be (Martínez-Priego and Romero-Iribas, 2021): (1) self-oriented emotions: such as satisfaction, wellbeing, or pleasure; (2) other-directed emotions: such as admiration, surprise, or envy (Michie and Gooty, 2005; Ortony et al., 1988); (3) other-oriented emotions: such as forgiveness, trust, or even (4) the so-called bonding feeling (characterized by accompanying gratuitous, generous, or selfless acts for the good of another). This classification leads to expanding the goals of ER to three levels: (1) hedonic (self-oriented emotions and other-directed emotions); (2) instrumental (some other-oriented emotions); and (3) eudaimonic, which include acts linked to the good of another (Ryff and Singer, 2002), as in bonding feelings.

## Methods

### Overview

This systematic review was conducted in accordance with the PRISMA guidelines (Moher et al., 2009). The PRISMA checklist is available in Supplementary Table 1. Given the main goal of this study, we adopted a flexible approach tailoring the PRISMA guidelines to the needs of this review. For example, formal quality assessment (including risk of bias, reporting bias, and certainty) was not carried out because the trustworthiness of the articles included did not directly relate to the overarching research aim, which aimed to identify the existence of emotion regulation models and theories across an extensive body of literature. This systematic review was pre-registered at Prospero platform (CRD42023491948).

### Search strategy

The electronic search was conducted by the review team in November 2023 to identify articles that have applied one or more emotion regulation models. The search for relevant articles entailed the use of Web of Science, Medline, and Scopus databases. These databases were chosen because they were expected to contain articles relevant to the field of emotion regulation across disciplines such as psychology, psychiatry, and behavioral sciences. The key search terms used were grouped into two different topics: Emotion Regulation and Models/Theories/Frameworks. Additional key informant consultation and manual search using reference lists from retrieved articles were also performed to identify further relevant papers of the main models. A full list of search terms can be found in Supplementary Table 2.

For this review, emotion regulation was defined as the ability to monitor, evaluate, and modify emotions to achieve goals (Thompson, 1994). Thus, emotion regulation includes both the strategies employed to regulate the emotions and the goals aimed to be achieve (Kopp, 1989). Furthermore, model/theory/framework were defined as any systematic approach used to understand, explain, or predict processes related to emotion regulation, including diverse theoretical options that target variables at different levels (Sovacool and Hess, 2017).

### Eligibility criteria

We only considered for the review those papers: (1) reporting one or more models/theories/frameworks of ER in healthy individuals (i.e., general population) or emotionally disordered individuals (i.e., psychopathological populations); (2) theoretical articles describing the ER model itself or empirical studies using an ER model (in which case we referred to the original article explaining the ER model); (3) published in English; (4) published in the last 5 years (from 2019 to 2023), although there will be no time restrictions for the referenced model/theory/framework they are using; (5) published in a peer-reviewed journal (i.e., gray literature, dissertations, books, and conference proceedings were excluded); (6) indexed in psychology, psychiatry or behavioral sciences; and (7) neuroscientific models exclusively focused on the biological foundations of ER, articles focused exclusively on a single ER strategy or goal, and the validation of scales based on previous ER models were excluded from the review.

### Identification and selection of studies

**Figure 1:**
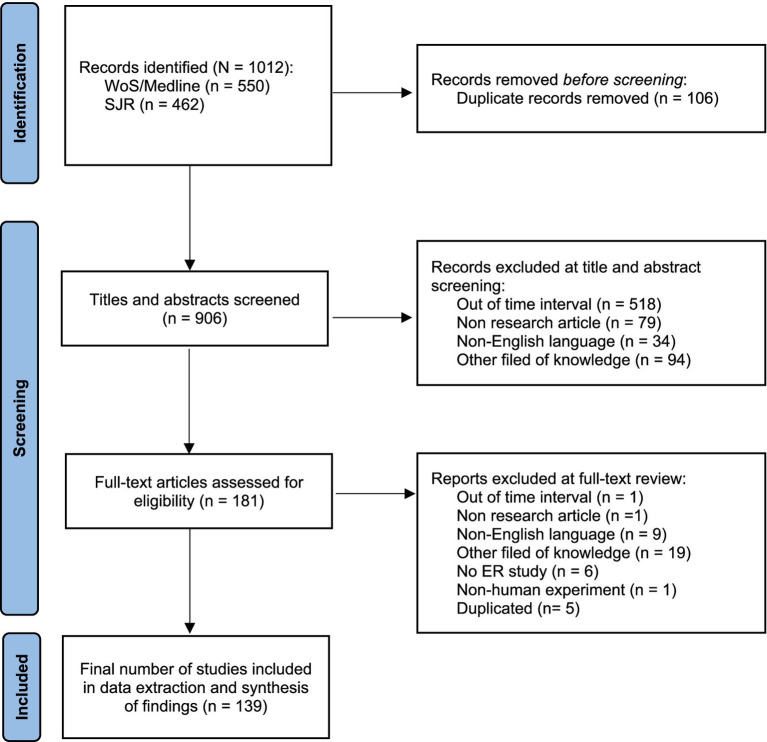
PRISMA flowchart of articles included and excluded.

The search was limited to articles published in the last 5 years (from 2019 to 2023) and yield 1,012 titles for initial consideration. Subsequently, all records were imported into Zotero software and reduced to 906 after removing duplicates. Papers then were title-checked to determine relevance to the research questions before undergoing further abstract screening by two independent reviewers (CM and BP) according to the eligibility criteria. Following two rigorous rounds of title and abstract screening, 181 full texts of all potentially eligible articles were examined and further screened by the reviewers. Articles that failed to meet the eligibility criteria were excluded and cross-checked the reasons for exclusion. Any conflicts in the decision making during the screening process were resolved via discussion with a third independent reviewer (PR) until consensus was reached. In total, 139 articles met the full eligibility criteria and were included for data extraction and synthesis. Figure 1 shows the PRISMA flowchart of articles included and excluded from the systematic review.

### Data extraction and synthesis

As the standardized extraction tools, such as Covidence or RevMan, did not meet the specific needs of this review, a modified extraction form and data synthesis was developed to include relevant characteristics to the research questions and the emotional regulation models reviewed (Supplementary Table 3). A two-step extraction process was carried out: (1) categorization of the articles according to the type of use of the ER model: analyze models, discuss models, propose models, review models, and use models/instruments; (2) in the case of empirical studies, we referred to the original article explaining the ER model. Then, two independent reviewers extracted the following outcomes from the ER models identified: (1) strategies used to regulate the emotions; and (2) goals that the person aims to achieve by regulating their emotions. Furthermore, additional outcomes were also extracted: (3) name of the emotion regulation model, theory, or framework; (4) general description of the model; and (5) instances of theory use (i.e., number of occurrences in which the theory was used).

On the one hand, the ER strategies of each model were classified based on three categories (see more details in Supplementary Table 4): (1) Cognitive strategies: referring to acts (not actions) aimed at modifying some cognitive process (e.g., thoughts, attention, memory, etc.). In this category, we would find strategies such as cognitive reappraisal, distraction, attentional shifting, or nonjudgmental awareness, among others; (2) Motivational-coping strategies: referring to elicited acts (from the Latin “elicere,” meaning to want or desire), which are voluntary and internal (without observable external manifestation). They are of a “conative” nature, meaning goal-oriented and motivational, but not behavioral (though they may extend into imperative acts). These acts have been termed “coping” in a dual sense: “effort” and “feeling capable” (Lazarus and Folkman, 1984). Both senses of coping precede potential behavior and influence evaluation (Scherer, 1982). In this category, we would find strategies such as setting meaningful goals aligned with personal values, emotion-focused coping, or resilience, among others; and (3) Behavioral strategies: referring to “imperative” (external) acts that modify behaviors and are observable by third parties. In this category, we would find strategies such as seeking social support, problem-solving, avoidance, or suppression, among others. As can be seen, the distinction between elicited and imperative acts helps avoid confusing acts of will such as hating or loving (elicited), with behaviors resulting from these acts such as violence or a caress (imperative). On the other hand, the ER goals and motives of each model were classified into three categories: (1) hedonic goals (e.g., I eat a cookie, *so I* feel better), (2) instrumental goals (i.e., contra-hedonic) (e.g., I delay a meal, *so I* will feel more attractive), and (3) eudaimonic goals (e.g., I give them a cookie as a gift, *so they* can satisfy their hunger).

## Results

The review identified 10 ER models. A general analysis of the articles included in the review was conducted (Table 1), revealing that approximately 46% of the reviewed articles use models or instruments related to ER models. Another 33.1% of articles are reviews of ER models; 10.8% of the articles propose an ER model, while 8.6% of the articles analyze models and 1.4% discuss various models in a more comprehensive manner.

**Table 1:** Categorization of the articles according to the type of use of the ER model.

Type of use	*N*	%
Analyze models	12	8.6%
Discuss models	2	1.4%
Propose models	15	10.8%
Review models	46	33.1%
Use models/instruments	64	46.0%

Within articles proposing ER models, we found that 93% of the research is based on the Process Model of Emotion Regulation (Gross, 1998). The remaining proposals are each based on different authors such as Bonano and Burton (2013), Festinger (1957), Lazarus and Folkman (1984), and Pekrun (2006). The only proposal we can consider as “new,” not based on any of the most referenced authors, is that of Mirzaie et al. (2022).

The Gross model is a comprehensive framework that conceptualizes ER as a process involving different strategies employed at different stages of emotional experience. It distinguishes between ER strategies applied before the emotional response is fully generated (i.e., antecedent-focused strategies), and those applied after the emotional response has occurred (i.e., response-focused strategies). The Gross model has evolved over time toward a more integrated view of ER, expanding from the initial focus on cognitive and behavioral strategies to include attentional strategies, along with the interaction between these strategies and the temporal dynamics of the emotion.

Table 2 presents the analysis of the ER models underlying each of the articles included in this review. Most of the articles are based, *explicitly*, on the Process Model of Emotion Regulation (Gross, 1998) (*n =* 87), followed at a considerable distance by other models such as Gratz and Roemer (2004) (*n =* 19), Garnefski et al. (2001) (*n =* 10), and Tamir et al. (2007) (*n =* 5). The latter, although also by Gross, incorporates some differences from the one proposed by this author in 1998, hence the decision to include it as a different model from the original.

**Table 2:** Explicit and implicit ER models underlying each of the articles included in this review.

Author of the model	Year	N° Explicit	N° Implicit
Gross	1998	87	19
Gratz and Roemer	2004	19	6
Garnefski	2001	11	–
Tamir and Gross	2007	5	–
Mayer and Solovey	1997	3	–
Ryan and Deci	2017	1	1
Mirzaie et al.	2022	1	–
Pekrun	2006	1	–
Eisenberg and Fabe	1992	1	–
Huges and Evans	2018	1	–
Lindsay and Creswell	2019	1	–
Lazarus and Folkman	1984	–	2
Festinger	1957	–	1
Bonanno and Burton	2013	–	1

Subsequently, an analysis was conducted of those articles referencing or proposing ER models to verify if their proposals were novel from the most commonly used ER models in the field (Table 2); that is, articles that *implicitly* are based on other models. It was observed that these less common ER models were, in turn, based on or inspired by the most popular models of Gross (1998) (*n =* 19), Gratz and Roemer (2004) (*n =* 6), and Lazarus and Folkman (1984) (*n =* 2) among others. Therefore, from this analysis, it is inferred that there are 15 models of Emotion Regulation that emerge from the systematic review and upon which a more detailed analysis will be carried out. From these 15, those referring to ER collaterally (e.g., those that primarily focus on other issues such as Emotional Intelligence) were excluded from further analysis (Eisenberg and Fabes, 1992; Hughes and Evans, 2018; Mayer and Salovey, 1997), or Mindfulness (Lindsay and Creswell, 2019). None of these articles contributes, uses, or is based on an ER model, and the ER is used as a moderator of Emotional Intelligence or a particular strategy (e.g., mindfulness).

The summary and classification of all ER models found in the review is provided in Table 3. As mentioned in the theoretical framework, the ER strategies of each model were classified based on three categories/dimensions of emotion understood as response, process, or state: (1) cognitive control strategies (e.g., cognitive reappraisal or distraction), (2) motivational-coping strategies (e.g., setting meaningful goals or emotion-focused coping), and (3) behavioral strategies (e.g., seeking social support or problem-solving). All models include cognitive strategies (*n =* 10), seven models also include coping strategies (Lazarus and Folkman, 1984; Garnefski et al., 2001; Pekrun, 2006; Bonano and Burton, 2013; Ryan and Deci, 2017; Mirzaie et al., 2022), and six models include behavioral strategies (Gross, 1998; Gratz and Roemer, 2004; Tamir et al., 2007; Mirzaie et al., 2022; Bonano and Burton, 2013; Lazarus and Folkman, 1984). Only 4 models include all three ER strategies (Bonano and Burton, 2013; Gratz and Roemer, 2004; Lazarus and Folkman, 1984; Mirzaie et al., 2022).

**Table 3:** Summary and classification of ER models.

Model	References	Description	Strategies	Strategies classification	Goals	Goal classification
Process Model of Emotion Regulation	Gross (1998)	Reappraisal as a central mechanism for ER, emphasizing hierarchical cognitive control structures.	Cognitive reappraisal, suppression, distraction, and acceptance, each influencing emotional outcomes differently.	*Cognitive and behavioral dimensions*	Enhancing wellbeing and homeostasis.	*Hedonic or instrumental*
Difficulties in emotion regulation	Gratz and Roemer (2004)	An integrative conceptualization of emotion regulation involves modulating emotional arousal, being aware of, understanding, and accepting emotions, and acting in desired ways regardless of emotional state. This model is proposed as the foundation for the DERS scale	Difficulties in acceptance, goal-directed behaviors, impulse control, emotional awareness, access to emotion regulation strategies, and emotional clarity.	*Cognitive, coping and behavior dimensions*	Adaptation and reducing distress.	*Hedonic or instrumental*
Cognitive emotion regulation	Garnefski et al. (2001)	ER model focused on 9 strategies that stem from the cognitive dimension of emotion. This model is proposed as the foundation for the CERQ scale.	Self-blame; acceptance; rumination; positive refocusing; refocusing on planning; positive reappraisal; putting into perspective; catastrophizing; and blaming others.	*Cognitive and coping dimensions*	Reducing distress and enhancing wellbeing-health.	*Hedonic or instrumental*
Implicit Theory of Emotions	Tamir et al. (2007)	How individuals’ implicit beliefs about emotions influence their ER strategies and outcomes.	Some strategies as (Gross, 1998): cognitive reappraisal, expressive suppression, distraction, acceptance, and rumination.	*Cognitive and behavioral dimensions*	Personal and social adjustment (homeostasis).	*Hedonic or instrumental*
Self-determination theory	Ryan and Deci (2017)	Individuals are intrinsically motivated to fulfill three basic psychological needs: autonomy, competence, and relatedness.	Fostering self-awareness, cultivating mindfulness, setting meaningful goals aligned with personal values, and practicing self-compassion.	*Cognitive and coping dimensions*	Fulfillment of basic psychological needs, which leads to wellbeing and optimal functioning	*Hedonic or instrumental*
Emotion regulation flexibility and electronic patient-reported outcomes	Mirzaie et al. (2022)	Broad notion of ER emphasizing the importance of flexibility and adaptability. Highlights the affect dynamic nature so the effectiveness of ER strategies may vary depending on the context and individual characteristics.	Reframing and reappraisal vs. resilience and tenacity; Suppressing vs. emotional disclosure and social support; distraction and attentional shifting vs. acceptance and tolerance; nonjudgmental awareness vs. problem-solving; inviting vs. activating positive emotion.	*Cognitive, coping and behavioral dimensions*	Promoting emotional wellbeing- health.	*Hedonic or instrumental*
Control Value Theory of Achievement Emotions	Pekrun (2006)	Emotions are influenced by perceptions of control over outcomes and the value attached to those outcomes, which in turn shape individuals’ emotional experiences during achievement-related activities.	Control appraisal and value appraisal.	*Cognitive and coping dimensions*	Optimizing emotional experiences during achievement pursuits.	*Hedonic or instrumental*
Transactional model of stress and coping	Lazarus and Folkman (1984)	Dynamic interaction between individuals and their environment, suggesting that stress arises from appraisals of the situational demands and one’s resources to cope with them.	Problem-focused coping (change or manage the stressor) and emotion-focused coping (regulating emotional responses through cognitive reappraisal or social support).	*Cognitive, coping and behavioral dimensions*	Reducing distress and enhancing adaptation to stressful situations.	*Hedonic or instrumental*
Cognitive dissonance theory	Festinger (1957)	Individuals experience psychological discomfort when they hold conflicting beliefs or engage in behaviors that contradict their attitudes or values.	Three mechanisms or strategies: modify the value of the elements, increase or decrease the weight of the elements, and alter the number of the elements.	*Cognitive dimension*	Reduce cognitive-behavioral dissonance.	*Hedonic or instrumental*
Regulatory flexibility model	Bonano and Burton (2013)	Individuals exhibit varying degrees of flexibility in adapting their regulatory responses based on situational demands and personal resources.	Reappraisal, suppression, distraction, problem-focused coping, repertoire of ER strategies are utilized flexibly, depending on sensitivity to external or internal feedback (contextual factors).	*Cognitive, coping and behavioral dimension*	Promoting adaptive coping and resilience in the face of adversity.	*Hedonic or instrumental*

On the other hand, the ER goals and motives of each model were classified into three categories: (1) hedonic goals, (2) instrumental goals (i.e., contra-hedonic), and (3) eudaimonic goals. The results showed that all reviewed ER models focus on hedonic and instrumental goals, with no model focusing explicitly on eudaimonic goals. However, the different ER models nuanced their ultimate goal differently: adaptation (*n =* 3), wellbeing-health (*n =* 2), wellbeing-homeostasis (*n =* 1), intra- and interpersonal homeostasis (*n =* 1), meeting psychological needs (*n =* 1), regulated response (*n =* 1), or reducing cognitive and behavioral dissonance (*n =* 1).

## Discussion

This systematic review summarizes and classifies the main strategies and goals advocated in current scientific literature on Emotion Regulation Models, theories, and frameworks. Furthermore, we explored the distinctions between the prevailing contemporary ER models, exemplified by the Gross Model, and classical conceptions of emotional dynamics, such as those proposed by Aristotle, Descartes, and Darwin.

The results of our study show that the underlying models of ER in the scientific literature are not always explicit (Sutton, 2004; Moumne et al., 2021), or they are limited to applying ER questionnaires without considering the limitations and biases that may introduce into the study being conducted. This is evident in 27% of the works, which simply use ER scales for evaluation, especially the Emotion Regulation Questionnaire (ERQ) (Gross and John, 2003) and the Difficulties in Emotion Regulation (DERS) (Gratz and Roemer, 2004). Furthermore, the uncritical inclusion of conceptual frameworks leads to excessively diverse conclusions regarding the effectiveness of ER interventions, as suggested by previous studies that reach opposing conclusions (Gross et al., 2019; Boemo et al., 2022). This same situation seems to repeat in our systematic review: only 7% of the articles contrast, compare, or review more than one ER model. However, the crux of this situation relates to the implications of choosing ER strategies and goals.

The results of this review suggest that the most widely employed model of ER is Gross’s model. With the cultivation of this emerging field, Gross hoped “to provide better answers than have ever before been possible to age-old questions about how emotions can-and should-be managed in order to optimize human functioning” (Gross, 1998, p. 288). Finally, his ER model focuses on the cognitive dimension of emotion, consisting of cognitive strategies (re-appraisal) that control and modify previous evaluations. Alongside this, hedonistic goals are established: “What are typical emotion regulatory goals? Individuals often seek to decrease negative emotions and increase positive emotions” (Gross, 1998, p. 286). A more comprehensive view of Gross’s model entails not forgetting that this author is aware of the multidimensionality of emotional response: “ER is denned and distinguished from coping, mood regulation, defense, and affect regulation” (Gross, 1998, p. 271). Distinguishing each of these dimensions can lead to an analytical view of the subject. However, the analytical consideration of the human being, by not aligning with reality, leads to theoretical and practical aporias (Polo, 2016). In contrast, the classical authors open up the possibility of establishing other places and resources for ER. Of particular interest is the inclusion of growth and pathways for the articulation of reason and emotion oriented toward a fuller life (Martínez-Priego and Romero-Iribas, 2024).

If we focus on ER strategies, as far as our study goes, all models appeal to the cognitive dimension as a key strategy for ER. This is consistent with the proposals of Aristotle (1984, 2018) [403a] and Descartes (1997). However, it differs from Darwin (1872) theory, where instinctive dynamics prevail over open human knowledge. Indeed, in the emotional process, the first moment is cognitive; even for James (1884), who cites Darwin when explaining that emotion is defined as the experience of body alterations. This body alteration is linked to a discriminative knowledge in which the stimulus adjusts to the subject like a key to a lock, instinctively. However, in the ER models reviewed, it is not always clear whether the rectification of the appraisal is merely control (as proposed by Descartes) or a hierarchy between levels of growth and improvement of knowledge of reality (as proposed by Aristotle). This second path of reappraisal implies that the knowledge rectifying the initial appraisal presupposes the first level of knowledge, part of it, and explicitly states its content; that is, it knows reality more accurately (Polo, 2015a, 2015b; Reyna-Fortes, 2024). Therefore, knowledge of reality prevails over narratives generated to improve only intrapersonal or interpersonal adjustment and, ultimately, the subject’s wellbeing (Baumeister et al., 2012). The subject’s narrative takes precedence over knowledge of reality.

If we adhere to Darwin’s proposal, we see that it remains relevant in current models. Indeed, adaptation, pleasure, or the reduction of imbalance states appear in all the ER models analyzed. Ultimately, these are homeostatic proposals (intra e interpersonal) regarding the individual. This presents a significant challenge: the improvement processes facilitated by ER lead to a terminal point, namely, achieving equilibrium. However, no human life has this structure, as growth must always continue (Joseph and Linley, 2006). Ceasing to develop organically does not mean that human growth has concluded (Vargas, 2019). At this point, it is worth emphasizing that the ER goals condition the outcomes of the intervention, as suggested by studies by Tamir et al. (2007). For this reason, it is important to distinguish between seeking homeostasis as subjective wellbeing and striving for eudaimonic wellbeing, where personal growth provides a broader source of resources and motivation that transcends overcoming particular obstacles. Without negating the importance of these, specific goals appear as means to achieve a better end. On the other hand, the eudaimonic end aligns with the actual potential for the person’s growth and enables the attainment of increasing states of happiness (Kristjánsson, 2018).

Regarding Descartes proposal, it is interesting to note the prevalence of “control” within the ER strategies employed in most models, which contrasts with some contemporary proposals that emphasize the importance of acceptance and mindfulness in ER processes (e.g., Roca et al., 2021, 2023). The underlying assumption of this dynamic can be twofold: (1) the hierarchical understanding of human operational capacities (from the sensory to the intellectual, and from coping to will); or (2) the existence of two instances: the mind and the body. The mind controls the body, either to increase pleasure or to carry out certain behaviors. However, neurophysiological studies can facilitate the psychosomatic articulation included in ER processes (Etkin et al., 2011; Etkin et al., 2015). That is, from the emotional brain and other physiological structures, it seems possible to show that there is no causal relationship between the cognitive and the behavioral (Martínez-Priego and Romero-Iribas, 2024). The presence of a causal view of some dynamics over others indicates that Cartesian dualism is still present in ER models.

The Aristotelian proposal suggests future lines of research to enrich ER models. On one hand, by explaining the psychosomatic nature of emotion, that is, the non-existence of causality between the mental and the corporeal. Thus, control would not be the main pathway for improving ER processes. On the other hand, it may allow for a deeper exploration of considering eudaimonic goal (virtue as real flourishing) as a criterion for human health, wellbeing, and growth. People’s beliefs are adjusted when confronted with reality rather than with the state of satisfaction or homeostasis. Lastly, the link between free acts and those motivated by emotional states connects knowledge with sound decision-making: this is what Aristotle himself called “phronesis” and is the subject of numerous academic works (De Caro and Vaccarezza, 2021; Kristjánsson and Fowers, 2024).

Lastly, regarding the ER goals, while people may give various reasons for the same behavior (e.g., not attending a party because I do not feel like it or because such events do not align with my values), the ER models analyzed understand that the goals are of an adaptive and homeostatic nature, either hedonic or instrumental, and do not include eudaimonic goals. Expanding ER goals to include eudaimonic components could alter the motivation behind ER, even when using the same strategies. For instance, a eudaimonic goal of suppressing emotions could be to hide disappointment over a gift because I value the other person’s intention more than my own satisfaction or pleasure from it. The emotional repertoire resulting from using one’s own emotion regulation resources could also be improved by including, for instance, other-oriented emotions and/or bonding feelings (Martínez-Priego and Romero-Iribas, 2021).

## Conclusion

In the present review, the most commonly used models in the current scientific literature have been highlighted (explicitly or implicitly). Among the ER models that emerged from the review, the Process Model of Emotion Regulation (Gross, 1998) stands out prominently as a hegemonic model in the field. Other models draw inspiration from theories stemming from Emotional Intelligence or Positive Psychology. There is a great deal of homogeneity among the proposed ER strategies and goals as well: the cognitive dimension takes precedence as the core of ER strategy, the regulation dynamic is governed by “control,” and the ER goals are hedonic or instrumental in nature.

While this review fulfills the proposed objectives, it also presents a series of limitations. For instance, more years could have been included in the analysis, not just the last five (although we aimed to focus on models used currently). Additionally, other databases could have been reviewed, and other disciplines interested in ER could have been expanded. The categorization of ER strategies and objectives of each model could be further detailed, which will be the subject of future research. Another limitation of our study is that we did not explore in detail the internal processes associated with each emotion regulation strategy and goal, even though these strategies and goals are applied at specific moments within the emotional process. Future studies should explore these internal processes more thoroughly to gain a deeper understanding of how these strategies and goals operate within the emotional process. Furthermore, future studies should consider exploring a broader range of articles, including those that focus on neuroscientific models of ER, articles focused on a single ER strategy or goal, and articles focused on the validation of scales based on previous ER models (e.g., Kraaij and Garnefski, 2019). This could help address gaps identified in our review and contribute to a more nuanced understanding of ER models across different contexts.

Although our study also has several strengths and practical implications, by addressing the complex task of reviewing and classifying the main strategies and goals advocated in the current scientific literature on ER Models, a crucial aspect for advancement in this field. Furthermore, we did not simply summarize contemporary ER models but analyzed them based on classical conceptions of emotional dynamics, such as those proposed by Aristotle, Descartes, and Darwin, which have guided and inspired current ER models. Modifying the aim of ER interventions allows for the comparison of longer-term expectations (motivations). Simultaneously, by proposing eudaimonic goals, the relative weight of short- and long-term objectives is redefined to align with reality. Empirical studies will need to consolidate this theoretically derived conclusion.

Both Descartes and Darwin are present in the analyzed ER models, Aristotle as well, as his proposal inaugurates the various areas of emotion study present to this day. However, some of their most significant contributions seem to be forgotten, such as the integration of the physical, operational, and growth dimensions within humans, as well as the view of psychological dynamics oriented towards an achievable goal through freedom: human growth.

## Data Availability

The original contributions presented in the study are included in the article/Supplementary material, further inquiries can be directed to the corresponding author.
